# Lack of Association between *Insulin Receptor Substrate2* rs1805097 Polymorphism and the Risk of Colorectal and Breast Cancer: A Meta-Analysis

**DOI:** 10.1371/journal.pone.0086911

**Published:** 2014-01-30

**Authors:** Yue Hu, Min Zhou, Kai Zhang, Xiangquan Kong, Xiaoyan Hu, Kang Li, Li Liu

**Affiliations:** 1 Cancer Center, Union Hospital, Tongji Medical College, Huazhong University of Science and Technology, Wuhan, China; 2 Department of Radiology, Union Hospital, Tongji Medical College, Huazhong University of Science and Technology, Wuhan, China; Loyola University Chicago, United States of America

## Abstract

**Background:**

Insulin receptor substrate-2 (IRS-2), a signaling adaptor protein, was involved in two cancer-related pathways (the phosphatidylinositol 3′-kinase (PI3K) and the extracellular signal-regulated kinase (ERK) pathways). Several studies have evaluated the association between *IRS2* rs1805097 (G>A) polymorphisms and the risk of colorectal and breast cancer. However, the results were inconsistent.

**Methodology/Principal Findings:**

A meta-analysis of seven published case-control studies (4 studies with 4798 cases and 5478 controls for colorectal cancer and 3 studies with 2108 cases and 2507 controls for breast cancer) were conducted to assess the strength of association using crude odd ratios (ORs) with 95% confidence intervals (CIs). For colorectal cancer, no obvious associations were found for all genetic models (homozygote comparison OR = 0.96, 95%CI 0.85–1.08, P_heterogeneity_ = 0.97; heterozygote comparison: OR = 0.91, 95%CI 0.73–1.13, P_heterogeneity_<0.01; dominant model: OR = 0.92, 95%CI 0.80–1.06, P_heterogeneity_ = 0.05; recessive model: OR = 1.02, 95%CI 0.91–1.14, P_heterogeneity_ = 0.60). In the subgroup analysis by ethnicity, control source and consistency of frequency with Hardy-Weinberg equilibrium (HWE), still no significant associations were observed. For breast cancer, also no obvious associations were found for all genetic models (homozygote comparison: OR = 0.95, 95%CI 0.71–1.26, P_heterogeneity_ = 0.10; heterozygote comparison: OR = 1.00, 95%CI 0.89–1.14, P_heterogeneity_ = 0.71; dominant model: OR = 0.98, 95%CI 0.87–1.10, P_heterogeneity_ = 0.55; recessive model: OR = 0.95, 95%CI 0.72–1.25, P_heterogeneity_ = 0.07). We performed subgroup analyses by sample size and did not find an association.

**Conclusions:**

This meta-analysis indicated that *IRS2* rs1805097polymorphism was not associated with colorectal and breast cancer risk.

## Introduction

Insulin receptor substrates (IRs) are signaling adaptor proteins consisting of six members (IRS-1-6) [1], [2]. Among the six family members, insulin receptor substrates 1 and 2(IRS-1and IRS-2) are the most thoroughly characterized members, owing to their wide tissue expression in rodents and humans [3]. IRS -2 shares significant structure with IRS -1, in that both proteins contain a N-terminal pleckstrin homology (PH)domain, phosphotyrosine binding (PTB) domains as well as a C-terminal tail consisting of numerous tyrosine and serine phosphorylation sites [4], [5]. The crucial role played by IRS-1 and IRS-2 in the regulation of insulin signaling was widely demonstrated by studies on knockout animal models. IRS-1 null mice showed growth retardation and mild resistance to insulin, but did not develop diabetes. IRS-2 null mice displayed metabolic defects in liver, muscle, and adipose tissues and they developed diabetes owing to pancreatic β-cell failure [6]. Recently, studies have shown they had a redundant role in mediating insulin action in hepatocytes. It was demonstrated that the deletion of both *IRS1* and *IRS2* genes in the liver of mice (L-DKO mice) prevented activation of hepatic Akt-Foxo1 phosphorylation and resulted in the development of diabetes [7], [8].

In addition, researchers have found out IRS-1 and IRS-2 mediate mitogenic and antiapoptotic signaling via binding to receptor tyrosine kinases (RKTs) such as ligand-phosphorylated insulin-like growth factor I receptor (IGF-IR) or insulin receptor (IR) [2], [9]. Moreover, considerable studies have revealed that this two signaling adaptors have themselves been shown to be transforming oncogenes [10]. *IRS1* plays a central role in cancer cell proliferation, in contrast, *IRS2* is associated with cancer cell motility and metastasis [9]. In addition, they are able to translocate into the nucleus and regulate transcription of genes involved in different stages of cancer progression [11]. Elizabeth et al reported that *IRS2* may be a driver oncogene in colorectal cancer and over-expressed IRS-2 activated the PI3 kinase pathway and increase cell adhesion [12]. Porter et al. and Chan et al. both figured out that a role for IRS-2 in cell migration rather than proliferation was shown in breast cancer [11], [13]. Also, Mathieu et al showed that deregulated expression of IRS-2 may contribute to liver tumor progression [14].

Until now, about 3644 single nucleotide polymorphisms (SNP) in the *IRS1* gene and 1704 SNP in the *IRS2* gene have been reported (http://www.ncbi.nlm.nih.gov/SNP), some of which have been shown as susceptible loci for several kinds of diseases, such as cancer and Type 2 diabetes mellitus (T2DM) [15]–[19]. For example, an important *IRS1* polymorphism rs1801278 (G>A) has been extensively investigated as a determinant of insulin resistance and a meta-analysis demonstrated that the A carriers significantly increased the risk of T2DM in those subjects whose mean age at diagnosis was less than 45 years [15], [17]. Furthermore, many epidemiological studies suggested this polymorphism affected the risk of many cancer types, including breast, colorectal, ovarian, prostate cancer and multiple myeloma [18], [20]–[23]. The *IRS2* gene is located on chromosome 13q34 [12]. The *IRS2* polymorphism rs2289046(A>G), which is a 3′UTR SNP, has already been reported it is closely related to the onset of pancreatic, breast and colorectal cancer [24]–[26]. Another *IRS2* rs1805097(G>A) polymorphism, a nonsynonymous SNP that was predicted to affect splicing, transcriptional regulation, and post-translational modification, is common [minor allele frequency (MAF)  = 0.30] (http://www.ncbi.nlm.nih.gov/SNP) and most frequently studied for the association with cancer risk, especially colorectal and breast cancer [18], [20], [21], [27]–[30]. In Chinese and northern Europe populations this polymorphism did not show associations with insulin sensitivity, insulin secretary function or T2DM, but in Italian and Asian Indian populations the variant allele may increase susceptibility to T2DM in obese people [16].

Although reported studies have focused on the association between the *IRS2* rs1805097(G>A) polymorphism and the risk of colorectal and breast cancer in diverse populations, the results remain inconclusive, partially due to the relatively small sample size in each of the published studies [30]. To confirm the effect of *IRS2* rs1805097 polymorphism on colorectal and breast cancer risk, a meta-analysis was performed.

## Methods

### Search Strategy

A literature search of Pubmed and EMbase was performed independently by two authors (updated to August 9, 2013), using the key words: ‘*Insulin receptor substrate2* or *IRS2*’, ‘polymorphism or variation’, ‘cancer or carcinoma or tumor or adenocarcinoma or neoplasm’. We evaluated all associated publications to retrieve the most eligible literature. Their reference lists were searched manually to identify additional eligible studies. When overlapping data of the same patient population were included in more than one publication, only the most recent or complete study was included. The results were limited to papers published in English.

### Inclusion Criteria and Exclusion Criteria

The following criteria were used for inclusion of the identified articles: (1) case-control studies for human, (2) investigation of the *IRS2* rs1805097 polymorphism and colorectal and breast cancer risk, (3) sufficient published data for estimating the OR and their corresponding 95%CI. Exclusion criteria included: (1) comment and review, (2) duplication of the previous publications, (3) no usable data reported.

### Data Extraction

Two investigators independently extracted data and reached consensus on all the items. For each eligible study, the following information was extracted: first author’s name, year of publication, ethnicity, cancer types, matching variables (age and sex), numbers of cases and controls, and genotype frequencies for cases and controls. Eligible studies were defined as population-based and hospital-based according to the control source. Different ethnicity descents were classified as Caucasian, Asian, and Mixed that included more than one ethnic descent. And when studies included subjects of more than one ethnicity and were able to separate, data were extracted separately for each ethnic group. We also classified a study as a large study if its total sample size was more than 1000, or it would be categorized as a small one.

### Statistical Analysis

We used the PRISMA checklist as protocol of the meta-analysis and followed the guideline (Checklist S1). HWE was firstly tested by Chi-square in each control group (P<0.05 was considered representative of statistical significance). The minor allele frequency (MAF) was also calculated for the controls. We calculated the OR and its 95%CI to assess the association between *IRS2* rs1805097 polymorphism and the risk of colorectal and breast cancer. Pooled ORs were computed for four genetic models of comparison: homozygote (AA vs GG), heterozygote (AG vs GG ), dominant (AA/AG vs GG), and recessive (AA vs AG/GG). A statistical test for heterogeneity was performed based on the I^2^ test and Q test [31], [32]. If I^2^>50% or P≤0.10which indicated heterogeneity in the comparison models among studies, so the estimated pooled ORs for each study were calculated using a random effects model (the DerSimonian and Laird method) [33]. Otherwise, the fixed-effects model was suitable (the Mantel–Haenszel method) [34]. Subgroup analyses were performed by ethnicity, source of control and consistency of frequency with HWE for colorectal cancer. For breast cancer, subgroup analyses were performed by sample size. Sensitivity analyses were also performed to identify the stability of the meta-analysis results.

### Evaluation of Publication Bias

Funnel plotting, in which the standard error of the log OR in each study was plotted against its log OR, was used to assess potential publication bias. An asymmetric plot suggested a possible publication bias. Funnel plot asymmetry was further assessed by the method of Egger’s linear regression test [35]. The significance of the intercept was determined by the t-test (P<0.05 was considered representative of statistically significant publication bias). The intercept provides a measure of asymmetry, and the larger its deviation from zero the more pronounced the asymmetry. We did not use Egger’s linear regression to test publication bias in a subgroup less than three studies.

All statistical tests were performed with the STATA software, version 12.0 (Stata Corporation, College Station, TX).

## Results

### Study Characteristics

**Figure 1:**
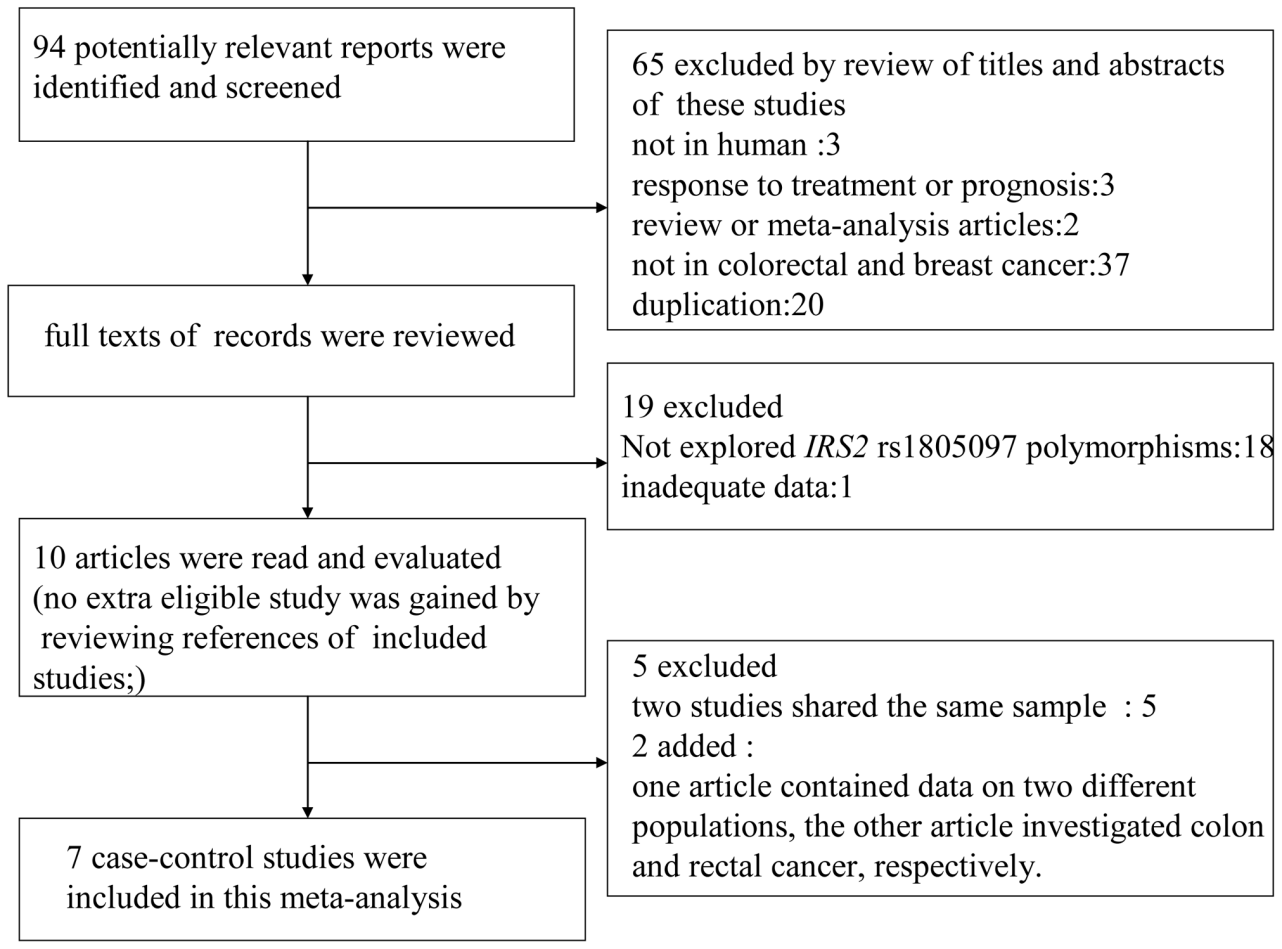
Flow diagram of the study selection process.

There were 94 articles identified by literature search from PubMed and EMBASE. The flow chart in Figure 1 summarized the selection process. In total, 10 articles were retrieved for further detailed evaluation [20], [21], [28]–[30], [36]–[40]. As shown in Figure 1, among them, 3 studies on colon and rectal cancer [36]–[38] and 2 studies on colon [39], [40] were excluded because of the overlapping data by the same authors. As 1 article presented the association in non-Hispanic white (NHW) and Hispanic populations, each study in the literature was considered separately [20]. 1 study investigated colon cancer and rectal cancer with the different controls, so we also treated them as separate studies [28]. At last, a total of 7 studies, including 4798 cases and 5478 controls for colorectal cancer and 2108 cases and 2507 controls for breast cancer, were used in the meta-analysis. All 7 studies were written in English. The 7 separate studies consisted of 4 Caucasian and 3 mixed ethnicity. There were 4 studies on colorectal cancer and 3 studies on breast cancer. Noticeably, deviation from HWE of genotype frequencies among the controls was detected in 2 studies [28]. Characteristics, genotypic frequencies (G/G, G/A, and A/A) and the MAF calculated for the controls of all individual studies (all were greater than 0.05) were listed in Table 1.

**Table 1:** Characteristics of studies included in the meta-analysis.

First author[Ref]	Year	Ethnicity^a^	Controlsource^b^	Matching	Cancer type	Case(genotype)			Control(genotype)				HWE^c^
						G/G	G/A	A/A	G/G	G/A	A/A	Maf^d^	
Pechlivanis[29]	2007	Caucasian	HB	Gender	Colorectal	211	277	81	268	309	106	0.38	0.281
Samowitz[21]	2006	Mixed	PB	Age, Gender	Colon	718	657	197	829	906	229	0.35	0.436
Slattery[28]	2005	Mixed	PB	Age, Gender	Rectal	325	255	195	420	304	260	0.42	<0.01
Slattery[28]	2005	Mixed	PB	Age, Gender	Colon	456	258	260	466	392	289	0.31	<0.01
Slattery[20]	2007	Caucasian(NHW)	PB	Age	Breast	497	546	130	544	594	190	0.37	0.178
Slattery[20]	2007	Caucasian(H)	PB	Age	Breast	212	264	99	262	347	117	0.4	0.906
Wagner[30]	2004	Caucasian	Un	Un	Breast	129	161	64	177	199	74	0.39	0.157

a NHW: Non-Hispanic White population; H: Hispanic population;

b HB: Hospital-based; PB: Population-based; Un: Unknown;

c HWE: Hardy-Weinberg equilibrium;

d MAF: minor allele frequencies.

### Meta-analysis Results

**Figure 2:**
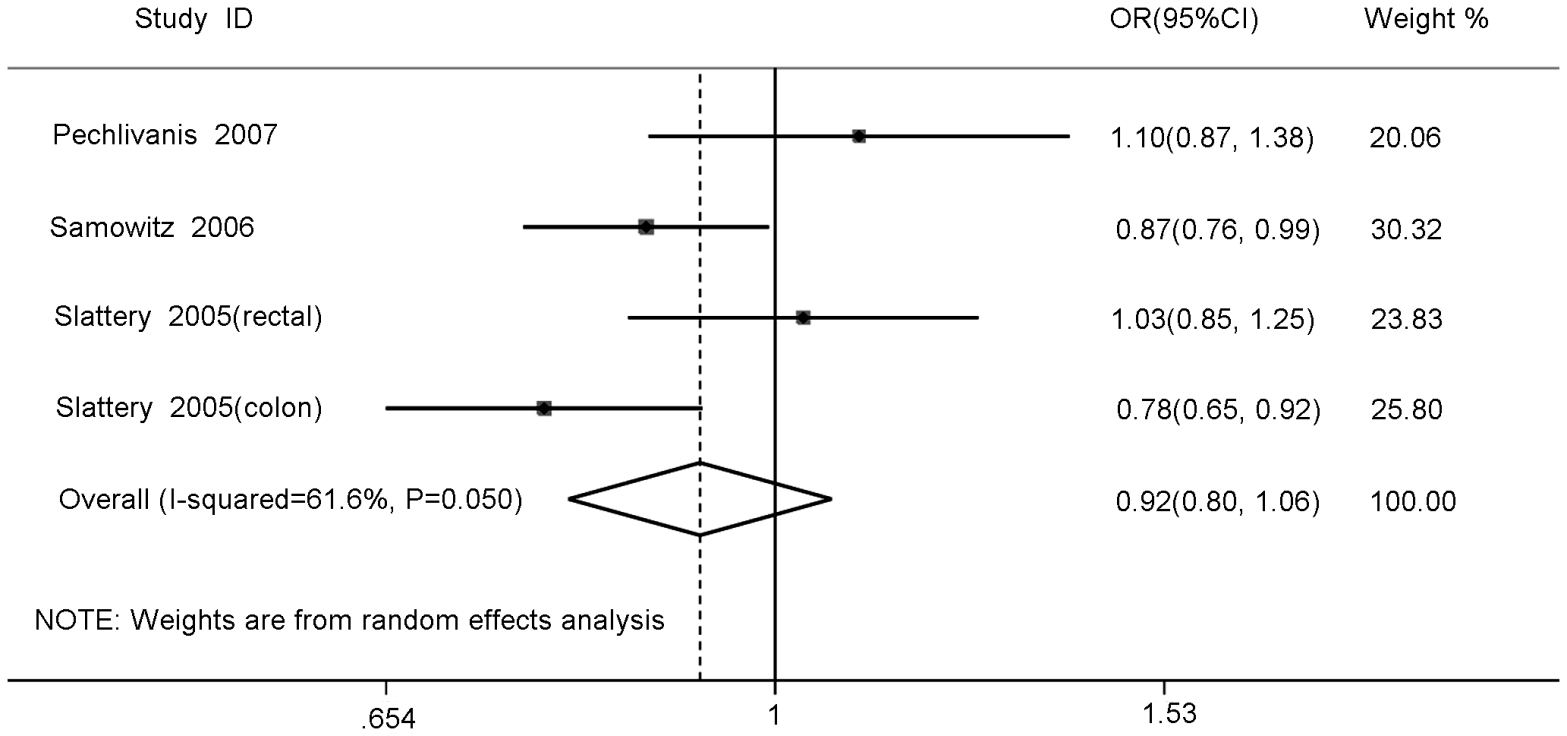
Forest plots for meta-analysis of the association between IRS2 rs1805097 polymorphism and colorectal cancer risk under dominant model (A/A+A/G vs. G/G).

**Figure 3:**
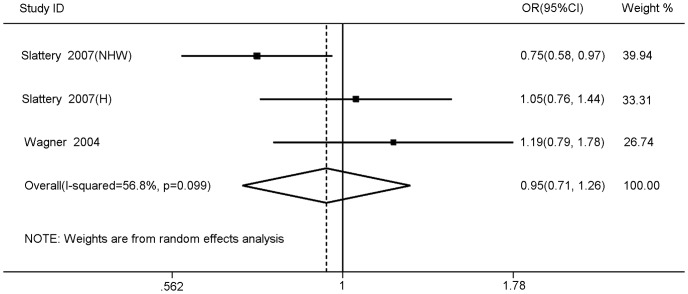
Forest plots for meta-analysis of the association between IRS2 rs1805097 polymorphism and breast cancer risk under homozygote model (A/A vs. G/G).

Table 2 showed the main results of this meta-analysis in details. Overall, no significant association between *IRS2* rs1805097 polymorphism and colorectal cancer risk was observed (homozygote comparison: OR = 0.96, 95%CI 0.85–1.08, P_heterogeneity_ = 0.97; heterozygote comparison: OR = 0.91, 95%CI 0.73–1.13, P_heterogeneity_<0.01; dominant model: OR = 0.92, 95%CI 0.80–1.06, P_heterogeneity_ = 0.05; recessive model: OR = 1.02, 95%CI 0.91–1.14, P_heterogeneity_ = 0.60) (Figure 2). Obvious heterogeneity was observed in the heterozygote and dominant comparisons. To determine the cause of heterogeneity among the studies and to obtain more accurate results, we conducted further meta-analyses stratified according to ethnicity, source of control and HWE. However, the heterogeneity still did not decrease. Similarly, the results of the reanalysis revealed that this polymorphism was not significantly associated with colorectal cancer risk. For breast cancer, also no obvious associations were found for all genetic models (homozygote comparison: OR = 0.95, 95%CI 0.71–1.26, P_heterogeneity_ = 0.10; heterozygote comparison: OR = 1.00, 95%CI 0.89–1.14, P_heterogeneity_ = 0.71; dominant model: OR = 0.98, 95%CI 0.87–1.10, P_heterogeneity_ = 0.55; recessive model: OR = 0.95, 95%CI 0.72–1.25, P_heterogeneity_ = 0.07) (Figure 3). In the homozygote and recessive models, we found there was obvious heterogeneity between studies. Then, we performed subgroup analysis by sample size to assess the source of the heterogeneity, but the results still did not change.

**Table 2:** Meta-analysis for the association between *IRS2* rs1805097 Polymorphism and Cancer Risk.

Genetic model	Comparisons	No. of studies	Test of association^a^			Test of heterogeneity	
			OR(95%CI)	p-value	Model	p-value	I^2^%
**Colorectal cancer**							
A/A vs. G/G	Overall	4	0.96(0.85–1.08)	0.51	F	0.97	0.0
	Mixed or PB	3	0.96(0.84,1.09)	0.52	F	0.88	0.0
	Caucasian or HB	1	0.97(0.69–1.37)	0.86	F	–	–
	HWE(yes)	2	0.99(0.82–1.18)	0.89	F	0.91	0.0
	HWE(no)	2	0.94(0.80–1.10)	0.45	F	0.74	0.0
A/G vs. G/G	Overall	4	0.91(0.73–1.13)	0.38	R	<0.01	79.9
	Mixed or PB	3	0.85(0.67–1.07)	0.17	R	<0.01	79.4
	Caucasian or HB	1	1.14(0.89–1.45)	0.29	F	–	–
	HWE(yes)	2	0.96(0.71,1.30)	0.79	R	0.03	78.3
	HWE(no)	2	0.85(0.53–1.36)	0.50	R	<0.01	89.7
A/A+A/G vs. G/G	Overall	4	0.92(0.80–1.06)	0.26	R	0.05	61.6
	Mixed or PB	3	0.88(0.76–1.02)	0.09	R	0.10	57.2
	Caucasian or HB	1	1.10(0.87–1.38)	0.44	F	–	–
	HWE(yes)	2	0.96(0.77,1.20)	0.70	R	0.09	66.0
	HWE(no)	2	0.89(0.68–1.18)	0.42	R	0.03	78.5
A/A vs. A/G+G/G	Overall	4	1.02(0.91–1.14)	0.73	F	0.60	0.0
	Mixed or PB	3	1.04(0.92–1.17)	0.55	F	0.54	0.0
	Caucasian or HB	1	0.90(0.66–1.24)	0.53	F	–	–
	HWE(yes)	2	1.03(0.87,1.22)	0.75	F	0.34	0.0
	HWE(no)	2	1.01(0.88–1.17)	0.86	F	0.33	0.0
**Breast cancer**							
A/A vs. G/G	Overall	3	0.95(0.71–1.26)	0.70	R	0.10	56.8
	Sample size (>1000)	2	0.87(0.63–1.21)	0.41	R	0.11	60.5
	Sample size (<1000)	1	1.19(0.79–1.78)	0.41	F	–	–
A/G vs. G/G	Overall	3	1.00(0.89–1.14)	0.95	F	0.71	0.0
	Sample size (>1000)	2	0.98(0.86,1.13)	0.82	F	0.65	0.0
	Sample size (<1000)	1	1.11(0.82–1.51)	0.51	F	–	–
A/A+A/G vs. G/G	Overall	3	0.98(0.87–1.10)	0.74	F	0.55	0.0
	Sample size (>1000)	2	0.95(0.84,1.08)	0.45	F	0.87	0.0
	Sample size (<1000)	1	1.31(0.85–1.51)	0.40	F	–	–
A/A vs. A/G+G/G	Overall	3	0.95(0.72–1.25)	0.70	R	0.07	61.8
	Sample size (>1000)	2	0.89(0.62–1.28)	0.53	R	0.05	73.1
	Sample size (<1000)	1	1.12(0.78–1.62)	0.54	F	–	–

a OR: odds ratio; R: random effect model; F: fixed effect model.

### Sensitivity Analysis and Bias Diagnostics

Funnel plots and Egger’s test were performed to assess publication bias of the literature. The results of Egger’s test are shown in Table 3. In our overall analysis, no evidence of publication bias was detected for both colorectal and breast cancer. Similar results were revealed by the shapes of the funnel plots (data not shown). A single study involved in the meta-analysis was deleted each time to reflect the influence of the individual data-set to the pooled ORs, and the corresponding pooled ORs were not materially altered except for heterozygote and dominant models in the subgroup of PB or mixed for colorectal cancer (data not shown).

**Table 3:** Egger’s linear regression test to measure the funnel plot asymmetric.

Comparisons	Y axis intercept^a^: (95%CI) p-value			
	A/A vs. G/G	A/G vs. G/G	A/A+A/G vs. G/G	A/A vs. A/G+G/G
**Colorectal cancer**				
Overall	0.30(−4.19,4.80)0.80	4.18(−20.10,28.47)0.54	4.64(−12.00,21.29)0.35	−2.99(−11.53,5.56)0.27
Mixed or PB	2.20(−74.00,78.40)0.78	1.53(−112.38,115.44)0.89	2.82(−95.17,100.80)0.78	−13.98(−101.89,73.94)0.29
**Breast cancer**				
Overall	6.41(−17.38,30.21)0.18	0.71(−22.58,24.00)0.77	2.33(−12.85,17.51)0.30	6.73(−39.42,52.88)0.32

a The significance of the intercept was determined using a t-test. P-values less than 0.05 were considered representative of publication bias.

## Discussion

Accumulating number of genetic association and genome-wide association studies (GWASs) have focused on the association between gene polymorphisms and cancer risk [41], [42]. However, the findings are generally inconsistent, probably due to some limitation in these studies such as small sample size. Meta-analysis is considered a powerful tool for summarizing the contradicting results from different studies with more statistical power, so that it can obtain more reliable results than a single study [43]. The findings suggested that the *IRS2* gene polymorphism was not significantly associated with both colorectal and breast cancer risk. In subgroup analysis, no significant association was observed. To the best of our knowledge, the present meta-analysis is the first article to assess the role of *IRS2* rs1805097 polymorphism in colorectal and breast cancer.

As one of the typical signaling adaptors, IRS-2 was involved in the phosphatidylinositol 3′ -kinase (PI3K) and the extracellular signal-regulated kinase (ERK) pathways, despite the fact it did not have intrinsic kinase activity and require upstream activators [10]. Additionally, IRS-2 could also interact integrins, hormones and cytokines such as IL-4 in a non-canonical manner by binding to cytoplasmic kinases(e.g.JAK) [11]. Owing to its presence in these important cancer-related pathways, IRS-2 was considered to be one of factors accelerating tumor progression and metastasis [3]. Jackson et al. reported that IRS-2 dependent signaling promoted cell motility and metastasis in human breast cancer cell [44].

An amino acid substitution of Gly to Asp at codon 1057 in *IRS2* gene by transversion of G to A was located close to two putative tyrosine phosphorylation sites at positions 1042 and 1072, and might change the tertiary structure and function of the protein [30], [45]. The relationship of the *IRS2* rs1805097 polymorphism to phenotypic features of insulin resistance, T2MD, polycystic ovary syndrome and several kinds of cancer has been intensively studied with controversial results. Several case-control studies found significant associations between *IRS2* rs1805097 polymorphism and gastric, colon and endometrial cancer [3], [28], [46]. However, these results were not confirmed by other studies [18], [29]. In this present meta-analysis, we retrieved 4 studies with 4798 cases and 5478 controls for colorectal cancer and 3 studies with 2108 cases and 2507 controls for breast cancer, no significant association was observed in all genetic models. Several factors contributing to the discrepancy between published studies might be different sample size, disease mechanisms or carcinogen exposure in different populations. There was a possibility that the *IRS2* variant genotypes may be tissue-specific. D’Alfonso et al. also found that the common *IRS2* rs1805097 variant did not appear to affect the level of IRS-2 expression and the ability to bind the P85 regulatory subunit of PI3-kinase [47]. Maybe this polymorphism was not functional but in linkage disequilibrium with a currently unrecognized functional polymorphism [48].

Certain potential limitations existed in our meta-analysis. Firstly, the number of published studies in our meta-analysis was insufficient, and the studies with small samples might decrease statistical power to properly evaluate the association. Secondly, when regarding with the ethnicity, most of the included studies conducted on Caucasians. Thus, it would be important to have more studies and samples from other ethnicity so that more accurate conclusions about the relationship between the *IRS2* rs1805097 polymorphism and colorectal and breast cancer risk might be determined. Thirdly, a more precise analysis could have been conducted, if individual data such as sex, body mass index, smoking and drinking status were available. Fourthly, besides a relatively high level of heterogeneity was detected in some comparisons, the influences of the individual data setting on the pooled ORs were significant in the subgroup of PB or mixed for colorectal cancer, which indicated the instability of the result. Therefore, the results should be interpreted with caution and confirmed from an additional analysis with more published studies in the furture. Fifthly, not all of studies are population-based. Owing to some types of unhealthy life styles or certain genotypes, controls in a hospital-based study might be susceptible to cancer. Therefore, controls selected from the healthy population were more representative for the general population, and might contribute more reliable results to a meta-analysis. Finally, gene-environment interactions should be considered in further studies if individual data of environmental exposure were available.

In conclusion, this meta-analysis suggests that the *IRS2* rs1805097 polymorphism may not contribute to the colorectal and breast cancer risk. However, large-sample studies of different ethnic groups with well matched cases and controls are necessary to further clarify the role of *IRS2* rs1805097 polymorphisms and these two types of cancer susceptibility in the future.

## Supporting Information

Checklist S1 **The guidelines of the preferred reporting items for systematic reviews and meta-analyses (PRISMA) statement.**
(DOC)
